# Contemporary Clinical Profile of Left-Sided Native Valve Infective Endocarditis: Influence of the Causative Microorganism

**DOI:** 10.3390/jcm12175441

**Published:** 2023-08-22

**Authors:** Gonzalo Cabezón, María de Miguel, Javier López, Isidre Vilacosta, Paloma Pulido, Carmen Olmos, Adrián Jerónimo, Javier B. Pérez, Adrián Lozano, Itzíar Gómez, J. Alberto San Román

**Affiliations:** 1 Instituto de Ciencias del Corazón (ICICOR), Hospital Clínico, Ciber de Enfermedades Cardiovasculares (CIBERCV), 47003 Valladolid, Spain; 2 Instituto Cardiovascular, Hospital Clínico San Carlos, 28040 Madrid, Spain; 3 Instituto de Investigación Sanitaria del Hospital la Princesa (IIS-IP), Hospital Universitario la Princesa, 28006 Madrid, Spain

**Keywords:** clinical profile, native valve infective endocarditis, microorganism, nosocomial

## Abstract

Studies focused on the clinical profile of native valve endocarditis are scarce and outdated. In addition, none of them analyzed differences depending on the causative microorganism. Our objectives are to describe the clinical profile at admission of patients with left-sided native valve infective endocarditis in a contemporary wide series of patients and to compare them among the most frequent etiologies. To do so, we conducted a prospective, observational cohort study including 569 patients with native left-sided endocarditis enrolled from 2006 to 2019. We describe the modes of presentation and the symptoms and signs at admission of these patients and compare them among the five more frequent microbiological etiologies. *Coagulase-negative Staphylococci* and *Enterococci* endocarditis patients were the oldest (71 ± 11 years), and episodes caused by *Streptococci viridans* were less frequently nosocomial (4%). The neurologic, cutaneous or renal modes of presentation were more typical in *Staphylococcus aureus* endocarditis (28%, *p* = 0.002), the wasting syndrome of *Streptococcus viridans* (49%, *p* < 0.001), and the cardiac in *Coagulase-negative Staphylococci*, *Enterococci* and unidentified microorganism endocarditis (45%, 49% and 56%, *p* < 0.001). The clinical signs agreed with the mode of presentation. In conclusion, the modes of presentation and the clinical picture at admission were tightly associated with the causative microorganism in patients with left-sided native valve endocarditis.

## 1. Introduction

Left-sided infective endocarditis (IE) bears a very poor prognosis, with in-hospital mortality rates ranging between 20 and 35% [1,2,3]. The identification of IE continues to be a challenge for physicians as usually, a high level of suspicion is necessary for making a diagnosis. The wide variability in the clinical presentation of IE, capable of involving each system of the organism, complicates and differs the diagnosis. Classical descriptions of the clinical course of IE are outdated and do not reflect the contemporary characteristics of IE because of the profound changes accounted for in this disease during the last decades [4,5,6,7,8]. Moreover, there are no studies comparing the clinical profile depending on the causative agent. As both the virulence of the microorganisms and the demographic characteristics vary widely with each type of IE, our hypothesis is that there are relevant differences in the clinical manifestations of IE depending on its etiology. The suspicion of the causative microorganism can be crucial to choose the best antibiotic treatment before its definitive identification and also in those cases where the etiology agent is not identified. Our goal is to describe the different modes of presentation of the disease and the clinical spectrum at admission for left-sided native valve IE in a wide contemporary series of patients and to compare the clinical profile among the most frequent etiologies.

## 2. Materials and Methods

### 2.1. Patient Population

This study was conducted at three tertiary care centers with surgical facilities, which have been working together on IE with the use of standardized protocols, uniform data collection and identical diagnostic and therapeutic criteria from the beginning of this study. All patients underwent a detailed clinical history, standard physical examination, 12 lead electrocardiograms, complete blood analysis, urine analysis, set of 3 blood cultures and transthoracic and transesophageal echocardiography at admission. An experienced multidisciplinary team composed of cardiologists, cardiac surgeons, experts on infectious diseases and microbiologists took care of these patients.

**Figure 1:**
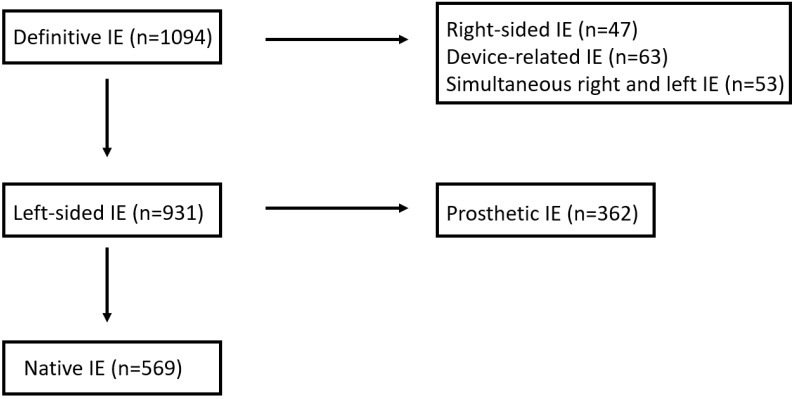
The selection process for patients with native LSIE.

From 2006 to 2019, 1094 episodes of IE were recruited and registered in an ongoing multipurpose database. The diagnosis of IE was based on the modified Duke criteria [9], and only definitive IE episodes were included in the study. The patient selection process is depicted in Figure 1. After the exclusion of right-sided IE (*n* = 47), device-related IE (*n* = 63) and IE with simultaneous involvement of left- and right-side valves (*n* = 53), 931 patients had exclusively left-sided IE. Finally, after the exclusion of prosthetic IE (*n* = 362), 569 were left-sided native valve IE and comprised our study group.

We analyzed 95 variables (28 demographic, 39 clinical, 3 radiologic, 8 electrocardiographic and 17 analytical), all of which were available within 24 h of the initial evaluation of patients with suspected IE. Comparisons among the 5 more frequent etiologies were performed.

### 2.2. Definition of Terms

Heart failure was diagnosed by an expert team according to the Framingham criteria [10]. Acute kidney failure was diagnosed as the presence of a serum creatinine > 2 mg/dL in patients without a previous diagnosis of chronic renal insufficiency; creatinine level deterioration > 50% in those with chronic renal insufficiency; and chronic anemia if serum hemoglobin was ≤13 g/dL in males or ≤12 g/dL in females for at least one year. Acute onset IE was applied when the time between the appearance of symptoms and hospital admission was <15 days. Uncontrolled infection was defined as the persistence of positive blood cultures for >7 days since adequate antibiotic treatment was established and/or the presence of perivalvular complication. For possible ports of entry of the infection, we considered those procedures with a risk of bacteremia performed within the previous 2 months before the onset of symptoms. The unidentified microorganism IE group comprised those IE cases without any microorganism identified using successive blood cultures, valve culture (if surgery was performed) and serologies for *Coxiella Burnetii*, *Chlamydia*, *Legionella*, *Mycoplasma*, *Brucella* and *Bartonella*. Referred cases were those patients transferred to the tertiary hospital from other healthcare centers where they were initially admitted. Clinical characteristics were recorded at the initial institution, and not at the tertiary center, irrespective if the patient was referred or not. Other characteristics of our protocol and the rest of the terms used in this study were defined elsewhere [11,12].

Table 1 shows the first signs and symptoms investigated with the patients classified into 8 modes of presentation, depending on the organs or systems involved: cardiac, wasting syndrome, rheumatic, neurologic, pulmonary, renal, cutaneous and digestive. These modes of presentation refer to the first clinical manifestations considered by the attending physician.

### 2.3. Statistical Analysis

Categorical variables are reported as absolute values and percentages and continuous variables are reported as the mean ± standard deviation or median [Q1–Q3]. Normal distribution of quantitative variables was verified with the Kolmogorov–Smirnov test. Qualitative variables were compared with the chi-squared test and Fisher’s exact test. Continuous variables were compared with an ANOVA or its equivalent nonparametric test, Kruskal–Wallis, for variables that were not normally distributed. A Bonferroni post hoc test was used for multiple comparisons. Statistical analysis was performed with IBM SPSS Statistics version 25 (IBM, Armonk, NY, USA). For all analyses, a two-tailed *p*-value of less than 0.05 was used to define statistical significance.

## 3. Results

### 3.1. Microbiological Profile

The microbiological profile for our series of left-sided native valve IE is as follows: 120 *Staphylococcus aureus* (SA) (21.1%), 112 *Streptococcus viridans* (SV) (19.7%), 76 *Enterococci* (EC) (13.4%), 56 *Coagulase-negative Staphylococci* (CNS) (9.8%), 45 other *Streptococci* (8%), 36 *Streptococcus gallolyticus* (6.3%), 26 *Gram-negative bacilli* (4.6%), 7 *Anaerobes* (1.2%), 6 fungi (1%), 27 *Polimicrobial* (4.7%), 45 unidentified microorganism (UM) (7.9%) and 13 others (2%). Of the SA-IE episodes, 18% of them were methicillin-resistant SA (MRSA).

### 3.2. Epidemiological Profile (Table 2)

The SV group was composed of younger patients than the SA, CNS and EC groups (mean age 59 years vs. 67, 71 and 71, respectively; *p* < 0.001). Suspected nosocomial origin was lower in the SV group than in the SA, CNS, EC, and UM groups (4% vs. 35%, 30%, 26% and 24%, respectively; *p* < 0.001. The time elapsed from the first symptom to admission was shorter in the SA group than in the SV, CNS, EC and UM groups (5 days vs. 30, 15, 21 and 12 days, respectively; *p* < 0.001).

Regarding the possible port of infection, dental manipulation was associated with SV rather than SA, CNS or EC (23% vs. 1%, 2% and 3%, respectively; *p* < 0.001), genitourinary manipulation was hardly exclusive of EC compared with SA, SV, CNS and UM (21% vs. 2%, 1%, 2% and 0%, respectively; *p* < 0.001) and indwelling intravascular catheters were more frequent in CNS compared with SV, EC and UM (36% vs. 2%, 3% and 11%l respectively; *p* < 0.001).

**Table 2:** Epidemiological profile of patients with left-sided native valve infective endocarditis and comparison between the 5 most frequent etiologies. *Co-n Staph* = *Coagulase-negative Staphylococci*. *S. aureus* = *Staphylococcus aureus*. *S. viridans* = *Streptococcus viridan*. IDUs = intravenous drug users. HIV: human immunodeficiency virus, COPD = chronic obstructive pulmonary disease.

	Total (*n* = 569)	*S. aureus* (*n* = 120) (a)	*S. viridans* (*n* = 112) (b)	*Co-n Staph.* (*n* = 56) (c)	*Enterococci* (*n* = 76) (d)	Unidentified (*n* = 45) (e)	*p*-Value	Post Hoc Comparison
Age (years), mean (SD)	66 ± 15	67 ± 13	59 ± 18	71 ± 11	71 ± 11	63 ± 15	<0.001	ab, bc, bd
Age > 70 years, *n* (%)	261 (46)	59 (49)	35 (31)	36 (64)	41 (54)	18 (40)	<0.001	ab, bc, bd
Males, *n* (%)	388 (68%)	76 (63)	79 (71)	39 (70)	57 (75)	33 (73)	0.463	
Referred, *n* (%)	302 (53)	71 (60)	62 (56)	29 (52)	42 (55)	19 (42)	0.370	
Nosocomial, *n* (%)	116 (20)	42 (35)	4 (4)	17 (30)	20 (26)	11 (24)	<0.001	ab, bc, bd, be
Acute onset, *n* (%)	274 (48)	98 (82)	22 (20)	29 (52)	29 (38)	22 (49)	<0.001	ab, ac, ad, bc, ae, bd, be
Days between first symptom and admission	32 [5–40]	5 [2–10]	30 [15–70]	15 [7–30]	21 [5–60]	12 [7–35]	<0.001	ab, ac, ad, ae
Antibiotics 15 days before admission, *n* (%)	152 (27)	34 (31)	29 (22)	20 (39)	15 (21)	19 (48)	0.041	de
Possible port of entry of the infection								
Unknown, *n* (%)	298 (52)	55 (46)	60 (54)	27 (48)	36 (47)	28 (62)	0.360	
Dental manipulation, *n* (%)	47 (8)	1 (1)	26 (23)	1 (2)	2 (3)	4 (9)	<0.001	ab, bc, bd
Gastrointestinal manipulation, *n* (%)	27 (5)	3 (3)	2 (2)	3 (5)	5 (7)	0 (0)	0.193	
Genitourinary manipulation, *n* (%)	24 (4)	2 (2)	1 (1)	1 (2)	16 (21)	0 (0)	<0.001	ad, bd, cd, de
Intravascular catheters, *n* (%)	77 (14)	33 (28)	2 (2)	20 (36)	2 (3)	5 (11)	<0.001	ab, ad, bc, cd, ce
Skin and soft tissue infections, *n* (%)	77 (14)	24 (20)	14 (13)	5 (9)	9 (12)	4 (9)	0.175	
Comorbid conditions								
Diabetes mellitus, *n* (%)	154 (27)	39 (33)	17 (15)	17 (30)	25 (33)	6 (13)	0.003	ab, bd
Cancer, *n* (%)	79 (14)	11 (9)	11 (10)	11 (20)	15 (20)	9 (20)	0.064	
Immunocomprimised state, *n* (%)	47 (8)	13 (11)	5 (5)	5 (9)	8 (10)	8 (18)	0.124	
Alcoholism, *n* (%)	57 (10)	9 (8)	12 (11)	3 (5)	7 (9)	7 (16)	0.427	
Chronic renal failure, *n* (%)	68 (16)	27 (23)	7 (6)	14 (25)	11 (15)	9 (20)	0.004	ab, bc
Hemodyalisis	21(3.7)	7(7)	2(2)	5(10.4)	2(3.1)	4(10.5)	0.123	
Colagenopathy, *n* (%)	15 (3)	5 (4)	0 (0)	2 (4)	1 (1)	3 (7)	0.110	
Chronic anemia, *n* (%)	129 (23)	25 (21)	17 (15)	24 (43)	22 (29)	12 (27)	0.002	ac, bc
COPD	49(8.6)	6(5)	2(1.8)	10(17.9)	10(13.2)	2(4.4)	0.001	bc, bd
Previous cardiopathy								
No cardiopathy, *n* (%)	288 (51)	65 (57)	52 (48)	18 (33)	37 (51)	26 (58)	0.056	
Rheumatic, *n* (%)	45 (8)	10 (8)	7 (6)	7 (13)	6 (8)	3 (7)	0.715	
Degenerative, *n* (%)	184 (32)	33 (28)	28 (25)	26 (46)	32 (42)	12 (27)	0.011	bc
Congenital, *n* (%)	40 (7)	7 (6)	15 (13)	3 (5)	1 (1)	7 (16)	0.008	bd, de
Myxoid, *n* (%)	37 (7)	3 (3)	14 (13)	2 (4)	4 (5)	3 (7)	0.027	ab
Hypertrophic cardiomyopathy, *n* (%)	14 (3)	3 (3)	1 (1)	4 (7)	1 (1)	1 (2)	0.151	
Previous endocarditis, *n* (%)	12 (2)	0 (0)	2 (2)	2 (4)	2 (3)	0 (0)	0.129	
IDUs, *n* (%)	8 (1)	2 (2)	3 (3)	0 (0)	1 (1)	1 (2)	0.785	
HIV, *n* (%)	8 (1)	3 (3)	2 (2)	0 (0)	2 (3)	0 (0)	0.624	

### 3.3. Modes of Presentation (Table 3)

Cardiac presentation of IE was less frequent in SV than in CNS, EC and UM (29% vs. 54%, 49% and 56%, respectively; *p* < 0.001) and similar to SA. Differences were found in SA compared with SV and EC presentation, with less wasting syndrome (14% vs. 49% and 34%, respectively; *p* < 0.001) but with more neurologic symptoms (28% vs. 13% and 8%, respectively; *p* = 0.002).

**Table 3:** Modes of presentation for patients with left-sided native valve infective endocarditis and a comparison between the 5 most frequent etiologies. *Co-n Staph* = *Coagulase-negative Staphylococci*. *S. aureus* = *Staphylococcus aureus*. *S. viridans* = *Streptococcus viridans*.

	Total (*n* = 569)	*S. aureus* (*n* = 120) (a)	*S. viridans* (*n* = 112) (b)	*Co-n Staph.* (*n* = 56) (c)	*Enterococci* (*n* = 76) (d)	Unidentified (*n* = 45) (e)	*p*-Value	Post Hoc Comparison
Cardiac, *n* (%)	214 (38)	36 (30)	32 (29)	30 (54)	37 (49)	25 (56)	<0.001	ac, ae, bc, bd, be
Wasting syndrome, *n* (%)	180 (32)	17 (14)	54 (49)	15 (27)	26 (34)	14 (31)	<0.001	ab, ad
Neurologic, *n* (%)	109 (19)	34 (28)	15 (13)	8 (14)	6 (8)	11 (24)	0.002	ab, ad
Rheumatic, *n* (%)	47 (8)	13 (11)	10 (9)	5 (9)	5 (7)	0 (0)	0.226	
Pulmonary, *n* (%)	44 (8)	5 (4)	5 (5)	5 (9)	6 (8)	5 (11)	0.375	
Renal, *n* (%)	48 (8)	19 (16)	8 (7)	2 (4)	6 (8)	4 (9)	0.063	
Digestive, *n* (%)	46 (8)	9 (8)	9 (8)	3 (5)	5 (7)	4 (9)	0.959	
Cutaneous, *n* (%)	34 (6)	13 (11)	9 (8)	0 (0)	2 (3)	1 (2)	0.019	

### 3.4. Clinical Characteristics at Admission (Table 4)

Stroke was less frequent in SV than in SA and UM (6% vs. 19% and 24%, respectively; *p* = 0.002). In addition, SV IE presented a lower rate of acute heart failure than CNS, EC and UM IE (23% vs. 54% and 49%, and 51%, respectively; *p* < 0.001). SA presented more septic shock compared with SV, CNS and EC (21% vs. 3%, 0% and 3%, respectively; *p* < 0.001). No significant difference between groups was found regarding perivalvular complications.

**Table 4:** Clinical signs and symptoms of patients with left-sided native valve infective endocarditis and comparison between the 5 most frequent etiologies. *Co-n Staph* = *Coagulase-negative Staphylococci*. *S. aureus* = *Staphylococcus aureus*. *S. viridans* = *Streptococcus viridans*.

	Total (*n* = 569)	*S. aureus* (*n* = 120) (a)	*S. viridans* (*n* = 112) (b)	*Co-n Staph.* (*n* = 56) (c)	*Enterococci* (*n* = 76) (d)	Negative (*n* = 45) (e)	*p*-Value	Post Hoc Comparison
Fever, *n* (%)	383 (67)	81 (69)	88 (79)	30 (54)	49 (65)	32 (73)	0.014	bc
Dyspnea, *n* (%)	205 (36)	36 (30)	30 (27)	28 (50)	33 (44)	24 (55)	0.001	ae, bc, be
Meningitis, *n* (%)	10 (2)	1 (1)	1 (1)	0 (0)	0 (0)	2 (4)	0.140	
Hemoptysis, *n* (%)	3 (0.5)	0 (0)	0 (0)	0 (0)	0 (0)	1 (2)	0.088	
Chest pain, *n* (%)	45 (8)	3 (3)	9 (8)	5 (9)	8 (11)	5 (11)	0.170	
Shivering, *n* (%)	226 (40)	44 (40)	53 (48)	23 (41)	27 (36)	16 (36)	0.428	
Confusional syndrome	66 (12)	24 (20)	9 (8)	3 (5)	4 (5)	8 (18)	0.003	ab, ad
Abdominal pain, *n* (%)	52 (9)	13 (11)	10 (9)	6 (11)	7 (9)	4 (9)	0.983	
Mialgia, *n* (%)	50 (8)	9 (8)	11 (10)	4 (7)	6 (8)	2 (4)	0.848	
Arthritis/spondylodiscitis, *n* (%)	46 (8)	13(11)	9(8)	5(9)	4(5)	0 (0)	0.180	
Cough, *n* (%)	56 (10)	3 (3)	16 (14)	4 (7)	6 (8)	8 (18)	0.006	ab, ae
Nausea, *n* (%)	39 (7)	9 (8)	5 (5)	4 (7)	5 (7)	2 (4)	0.864	
Headache, *n* (%)	31 (5)	2 (2)	7 (6)	4 (7)	3 (4)	4 (9)	0.251	
Stroke, *n* (%)	77 (14)	23 (19)	7 (6)	5 (9)	6 (8)	11 (24)	0.002	ab, be
Ischemic, *n* (%)	60 (78)	17 (74)	5 (71)	5 (100)	6 (100)	9 (82)	0.446	
Hemorrhagic, *n* (%)	17 (22)	6 (26)	2 (29)	0 (0)	0 (0)	2 (18)		
Splenomegaly, *n* (%)	38 (7)	3 (3)	13 (12)	1 (2)	3 (4)	2 (5)	0.015	ab
Cutaneous lesions, *n* (%)	51 (9)	21 (18)	10 (9)	1 (2)	4 (5)	7 (16)	0.006	ac
Osler nodes, *n* (%)	11 (1.9)	7 (33)	2 (20)	0 (0)	1 (25)	0 (0)	0.428	
Janeway lesions, *n* (%)	18 (3.2)	11 (53)	3 (30)	0 (0)	1 (25)	3 (43)	0.539	
Splinter hemorrhages, *n* (%)	33 (5.8)	16 (76)	4 (40)	0 (0)	4 (100)	6 (86)	0.029	
New murmur, *n* (%)	253 (45)	34 (30)	59 (53)	27 (48)	45 (59)	19 (43)	<0.001	ab, ad
Acute heart failure, *n* (%)	211 (37)	38 (32)	26 (23)	30 (54)	37 (49)	23 (51)	<0.001	ac, bc, bd, be
Septic shock	44 (8)	25 (21)	3 (3)	0 (0)	3 (3)	3 (7)	<0.001	ab, ac, ad
Perivalvular complication, *n* (%)	146 (26)	28 (23)	39 (35)	20 (36)	19 (25)	14 (31)	0.234	

## 4. Discussion

Clinical suspicion is the initial step in the diagnosis of IE, which sometimes can be exceedingly difficult because IE can mimic many diseases by involving any organ of the body. The changes involving the demography of IE patients, lifestyle, cardiovascular care and antibiotic progress have probably contributed to the evolution of the current clinical manifestations with respect to classical descriptions. As an example, nowadays, IE patients have increasing age and comorbidities [7], rheumatic disease is promptly treated and the widespread use of antibiotics has stimulated the selection of drug-resistance bacteria.

The typical Oslerian clinical picture consisting of fever, heart murmur, splenomegaly and cutaneous manifestations is very rarely seen nowadays, as reflected in our series (six patients, 1%). From a practical point of view, the diagnosis of IE should be considered in any patient who has an unexplained fever, especially if the patient has organ involvement attributable to an embolic phenomenon or presents a multisystem disease.

Several asseverations from our results have to be underlined: (1) identifying a possible port of entry may help to determine the causative microorganism, (2) not only SA but also less aggressive microorganisms frequently affect normal valves and (3) the modes of presentation and the clinical picture are tightly associated with the microorganism. All these findings deserve to be commented on.

Firstly, an essential part of the clinical history when facing patients with IE is addressing possible ports of entry. Our results reinforce that the relationship between *Staphylococci* and intra-vascular catheters, and *Enterococci* with genitourinary manipulations seen in classical works [13,14,15], remains valid for the current series. Whether dental manipulation and SV IE are associated is still a matter of debate to which our study cannot respond. Despite there is a strong association between dental manipulation and SV IE in our series, a cause-effect relationship cannot be established.

It is well known that virulent organisms (SA) have high-adherence properties and infect valves with minor underlying lesions or even normal valves [16,17]. However, it is worth mentioning that half of the patients infected with SV did not have previously known valvular disease. This fact has been already pointed out by other authors. Castillo et al. found that the proportion of patients with IE affecting normal valves increased by 128% during the last years [18]. From a clinical point of view, it is important that half of the patients with IE will not have a previous history of valvulopathy—one of the minor diagnostic criteria of IE [9]—making the diagnostic even more difficult.

To our knowledge, this is the first work to address a putative relationship between the mode of presentation and the most frequent causative agents. Although classically explained in old textbooks, assembling different symptoms affecting the same organs or systems in modes of presentation is not routinely performed in daily practice. Our results encourage this initial approach for patients with IE aiming at foreseeing the etiology of the disease. From a practical standpoint, empiric antibiotic treatment before receiving blood cultures results may be driven, at least partially, by the mode of presentation.

SA IE, the leading cause of native valve IE in our series, is clinically expressive and bears symptoms suggestive of severe infection and systemic involvement: acute onset, confusional syndrome, cutaneous lesions, acute renal failure and septic shock. As well, SA-IE carries a high rate of septic shock and neurologic symptoms, a fact stated before in previous publications [19]. This wide group of early manifestations is probably the reason why acute heart failure was not especially suggestive of SA at admission, despite its known capability to provoke cardiac damage. As antibiotic resistance is a major concern and it is increasing with time [20], it is remarkable that the observed incidence of MRSA-IE in our population constitutes 18% of all SA-IE, a similar percentage to other series published a decade ago [19].

The characteristic clinical profile of IE caused by SV is a subacute presentation, with unspecific symptoms starting weeks before admission and affecting younger ambulatory patients. The virulence of SV is low, and it is common to make the diagnosis late in the course of the disease, as 54% of patients had compatible manifestations more than 1 month before diagnosis. The subacute manifestations justify lower rates of acute heart failure and stroke at admission than other microorganisms.

CNS-IE affects predominantly patients with previous cardiopathy—mainly degenerative—who have a history of indwelling intravascular catheters. Avoiding unnecessary intravenous lines, providing scrupulous asepsis during catheter implantations, revising sites of punctures daily and rapidly withdrawing infected intravenous catheters could prevent CNS-IE. The presentation of this type of endocarditis was acute in 52% of the cases; there was cardiac involvement in half of the patients, and renal failure was present in almost one-third of them.

Similar to SV-IE, native valve IE due to enterococci is typically subacute and gives rise to wasting syndrome manifestations. More than one-third of the patients did not present fever at admission despite only 21% of the patients being under antibiotics within the previous 15 days.

Finally, left-sided native valve UM-IE has “intermediate” characteristics, probably reflecting the fact that this group is composed of patients with decapitated infections because of the widespread use of antibiotics before the diagnosis (48%). Half of the patients had cardiac manifestations at admission, which was the highest among the five etiologies analyzed in this study.

This work provides a detailed clinical description of a contemporary series of left-sided native valve IE and compares, for the first time, the clinical profile among the most frequent causative microorganisms. We not only described the percentages of presentation of the most important signs or symptoms, which is the most common way of approaching this issue [21], but also emphasized the modes of presentation depending on the organ or systems clinically involved, which is more useful from a practical point of view.

We must acknowledge some limitations of our study. The three participant centers are tertiary, and approximately half of our patients were referred from other institutions. Patients with both a good clinical evolution who do not need surgery and those with a very rapid and fatal course may be not referred to our centers, and thus, their clinical profile has not been recorded. Cardiac, renal and cutaneous clinical presentation at admission in referred patients tended to be more frequent than in non-referred patients.

There were no differences in the microorganism responsible for IE between referred and non-referred patients. This is an inherent bias of all the series, albeit we consider that the results are only applicable to tertiary centers. Clinical characteristics of the referred patients are obtained at admission in the first hospital that a patient attended and not upon arrival to the tertiary center. This was performed using direct anamnesis and exhaustive revision of clinical history and data collected at admission in the referring hospital.

In conclusion, the modes of presentation and the clinical picture at admission were tightly associated with the causative microorganism in patients with left-sided native valve endocarditis. The scientific community should continue to make efforts to characterize left-sided IE in the most accurate fashion, as it is an evolving disease throughout the years.

## Figures and Tables

**Table 1:** Correspondence between signs and symptoms and modes of presentation.

Mode of Presentation	Signs and Symptoms
Cardiac	Acute heart failure, new heart murmur, atrioventricular block
Wasting syndrome	Weight loss, asthenia, anorexia
Neurologic	Stroke (ischemic or hemorrhagic), confusional syndrome, meningeal syndrome
Rheumatic	Arthralgia, myalgia, arthritis, spondylitis
Pulmonary	Pneumonia, cough, pulmonary embolism, hemoptysis
Renal	Renal failure, proteinuria, hematuria, flank pain
Digestive	Abdominal pain
Cutaneous	Osler nodes, Janeway lesions, splinter hemorrhages, petechiae

## Data Availability

The data presented in this study are available on request from the corresponding author. The data are not publicly available due to privacy aspects.

## References

[1] Thuny F., Grisoli D., Collart F., Habib G., Raoult D. (2012). Management of infective endocarditis: Challenges and perspectives. Lancet.

[2] Cahill T.J., Baddour L.M., Habib G., Hoen B., Salaun E., Pettersson G.B., Schäfers H.J., Prendergast B.D. (2017). Challenges in Infective Endocarditis. J. Am. Coll. Cardiol..

[3] Habib G., Erba P.A., Iung B., Donal E., Cosyns B., Laroche C., Popescu B.A., Prendergast B., Tornos P., Sadeghpour A. (2019). EURO-ENDO Investigators. Clinical presentation, aetiology and outcome of infective endocarditis. Results of the ESC-EORP EURO-ENDO (European infective endocarditis) registry: A prospective cohort study. Eur. Heart J..

[4] Hermans P.E. (1982). The clinical manifestations of infective endocarditis. Mayo Clin. Proc..

[5] Fernández-Hidalgo N., Tornos P. (2013). Epidemiology of infective endocarditis in Spain in the last 20 years. Rev. Esp. Cardiol..

[6] Ambrosioni J., Hernandez-Meneses M., Téllez A., Pericàs J., Falces C., Tolosana J., Vidal B., Almela M., Quintana E., The Hospital Clinic Infective Endocarditis Investigators (2017). The Changing Epidemiology of Infective Endocarditis in the Twenty-First Century. Curr. Infect. Dis. Rep..

[7] Sevilla T., López J., Gómez I., Vilacosta I., Sarriá C., García-Granja P.E., Olmos C., Di Stefano S., Maroto L., San Román J.A. (2017). Evolution of prognosis in left-sided infective endocarditis: A propensity score analysis of 2 decades. J. Am. Coll. Cardiol..

[8] Vincent L.L., Otto C.M. (2018). Infective Endocarditis: Update on Epidemiology, Outcomes, and Management. Curr. Cardiol. Rep..

[9] Li J.S., Sexton D.J., Mick N., Nettles R., Fowler V.G., Ryan T., Bashore T., Corey G.R. (2000). Proposed modifications to the Duke criteria for the diagnosis of infective endocarditis. Clin. Infect. Dis..

[10] McKee P.A., Castelli W.P., McNamara P.M., Kannel W.B. (1971). The natural history of congestive heart failure: The Framingham study. N. Engl. J. Med..

[11] López J., Revilla A., Vilacosta I., Sevilla T., Villacorta E., Sarriá C., Pozo E., Rollán M.J., Gómez I., Mota P. (2010). Age-dependent profile of left-sided infective endocarditis: A 3-center experience. Circulation.

[12] Graupner C., Vilacosta I., SanRomán J., Ronderos R., Sarriá C., Fernández C., Mújica R., Sanz O., Sanmartín J.V., Pinto A.G. (2002). Periannular extension of infective endocarditis. J. Am. Coll. Cardiol..

[13] Dahl A., Bruun N.E. (2013). Enterococcus faecalis infective endocarditis: Focus on clinical aspects. Expert. Rev. Cardiovasc. Ther..

[14] Fernández-Hidalgo N., Almirante B., Tornos P., Pigrau C., Sambola A., Igual A., Pahissa A. (2008). Contemporary epidemiology and prognosis of health care-associated infective endocarditis. Clin. Infect. Dis..

[15] Chu V.H., Woods C.W., Miro J.M., Hoen B., Cabell C.H., Pappas P.A., Federspiel J., Athan E., Stryjewski M.E., Nacinovich F. (2008). Emergence of coagulase-negative staphylococci as a cause of native valve endocarditis. Clin. Infect. Dis..

[16] Hoerr V., Franz M., Pletz M., Diab M., Niemann S., Faber C., Doenst T., Schulze P., Deinhardt-Emmer S., Löffler B.S. (2018). *aureus* endocarditis: Clinical aspects and experimental approaches. Int. J. Med. Microbiol..

[17] Galar A., Weil A.A., Dudzinski D.M., Muñoz P., Siedner M.J. (2019). Methicillin-Resistant *Staphylococcus aureus* Prosthetic Valve Endocarditis: Pathophysiology, Epidemiology, Clinical Presentation, Diagnosis, and Management. Clin. Microbiol. Rev..

[18] Castillo J.C., Anguita M.P., Ruiz M., Peña L., Santisteban M., Puentes M., Arizón J.M., de Lezo J.S. (2011). Changing epidemiology of native valve infective endocarditis. Rev. Esp. Cardiol..

[19] Abdallah L., Remadi J.-P., Habib G., Salaun E., Casalta J.-P., Tribouilloy C. (2016). Long-term prognosis of left-sided native-valve *Staphylococcus aureus* endocarditis. Arch. Cardiovasc. Dis..

[20] Lakhundi S., Zhang K. (2018). Methicillin-Resistant *Staphylococcus aureus*: Molecular Characterization, Evolution, and Epidemiology. Clin. Microbiol. Rev..

[21] Murdoch D.R., Corey G.R., Hoen B., Miró J.M., Fowler V.G., Bayer A.S., Karchmer A.W., Olaison L., Pappas P.A., Moreillon P. (2009). Clinical presentation, etiology, and outcome of infective endocarditis in the 21st century: The International Collaboration on Endocarditis-Prospective Cohort Study. Arch. Intern. Med..

